# Flux profile at focal area of concentrating solar dishes

**DOI:** 10.1038/s41598-021-03768-w

**Published:** 2021-12-28

**Authors:** M. Ebrahim Foulaadvand, Amir Aghamohammadi, Parvin Karimi, Hadi Borzouei

**Affiliations:** 1 Department of Physics, Faculty of Science, University of Zanjan, Zanjan, PC 45371-38791 Iran; 2 Department of Physics, Faculty of Physics and Chemistry, Alzahra University, Tehran, Iran; 3 Department of Physics, South Tehran Branch, Islamic Azad University, Tehran, Iran; 4 Campus of New Technologies, Department of Engineering Sciences, Hakim Sabzevari University, Sabzevar, Iran

**Keywords:** Optical physics, Solar thermal energy

## Abstract

We analytically, experimentally and computationally explore the solar radiation flux distribution in the interior region of a spherical mirror and compare it to that of a paraboloidal one with the same aperture area. Our investigation has been performed in the framework of geometrical optics. It is shown that despite one can assign a quasi focus, at half the radius, to a spherical mirror, the light concentration occurs as well on an extended line region which starts at half-radius on the optical axis. In contrast to a paraboloidal concentrator, a spherical mirror can concentrate the radiation parallel to its optical axis both in a point-focus and in a line-focus manner. The envelope of the reflected rays is also obtained. It is shown that the flux distribution has an axial symmetry. The radial dependence of the flux on a flat circular receiver is obtained. The flux longitudinal dependence is shown to exhibit three distinctive regions in the interval [0, *R*] (R is mirror radius). We obtain the radiational (optical) concentration ratio characteristics and find the optimal location of the flat receiver of a given size at which the concentration ratio is maximised. In contrast to a parabolic mirror, it is shown that this location depends on the receiver size. Our findings offers that in spherical mirrors one can alternatively use a line receiver and gains a considerable thermal energy harvest. Our results are supported by Monte Carlo ray tracing performed by Zemax optical software. Experimental validation has been performed in lab with a silver-coated lens as the spherical mirror.

## Introduction

The use of renewable energy sources and replacement of traditional fossil-based fuel resources by the renewable ones is rapidly spreading worldwide^1^. Growing energy demands and the threats of global warming have pushed many governments to invest a notable amount of financial resources on this extremely important issue^1–3^. An abundant renewable energy source is sunlight and during past decades especially after oil crisis in 1973 serious attempts were initiated in utilising the sunlight as a source of clean energy. The solar energy is one of the cost effective paths to displace fossil fuels. It can be harvested and converted into energy by two paradigms of technicality: photovoltaic and Concentrated Solar Power (CSP)^4,5^. In CSP technology the harvest of solar irradiation is attained by solar collectors^6^. These devices can concentrate solar radiation and convert it into low, medium or high temperature useful heat that can be used in power generation, thermal processes, desalination, solar fuel production and many other civil and industrial applications^5^. A solar concentrating collector operates by focusing the solar radiation onto a small focal area. Parabolic troughs and dish concentrators are two main classes of such concentrators^4,6,7^. A parabolic trough focuses the sunlight onto a focal line and is a generic example of line-focus systems. On the hand, a dish concentrator (normally a parabolic dish) focuses the sunlight beam on a focal area (theoretically on a point) and is regarded as a point-focus system in the literature^6,8^. Aside from the degree of concentration, for practical purposes, it is significantly important to optimally design a receiver and its location/orientation in the focal area for an optimal absorptance of the concentrated solar radiation^9–15^. This is not possible unless a detailed knowledge of the radiative flux distribution is analytically or numerically available. In this paper our study is targeted on a spherical concentrator. The ideal point-focus collector is a paraboloidal dish. An ideal paraboloidal dish can concentrate the solar radiation on a point known as the *focus*. However, in practice construction of a paraboloidal dish is not an easy task especially when the size of the dish becomes large^16,17^. From engineering viewpoint fabricating a large sheet of a parabolic reflector is a cumbersome problem. In order to circumvent this problem, CSP experts have proposed alternative ideas. Some researchers have proposed a faceted reflecting surface instead of a single surface^18^. Others have suggested using an array of small flat mirrors assembled on a parabolic-like curved supporting structure^19–22^. In^24^ ray-tracing simulation has been implemented and it was demonstrated that the optimal design is a paraboloidal structural envelope with spherical facets mounted on it. It seems evident that there is a practical interest in solar thermal engineering to use spherical mirrors or facets instead of paraboloidal ones as alternatives or approximates^12,24,25^. This paper provides a detailed mathematical investigation of the spatial distribution of radiative flux in a spherical concentrating mirror. We will also compare the optical performance of a spherical mirror with its counterpart a paraboloidal mirror. Mathematical analysis of this study may shed more light on the path of solar thermal engineers for a better conversion of sunlight thermal energy to useful heat and can strengthen the connection between physics and solar thermal engineering communities. The paper's organisation is as follows: in “Spherical mirror” section, we obtain the thermal flux distribution in the interior of a spherical mirror. In “Ray tracing Monte Carlo simulation” section the simulation results are presented and compared to analytic ones. In “Experiment and empirical data” section we present our empirical data from the lab experiment. In “Concentration ratio” section there is a discussion on the concept of concentration ratio and we obtain the receiver optimal location for maximising this ratio. In “Comparison to parabolic dish” section, we compare the optical characteristics of the spherical mirror with a paraboloidal one. In “Sun angular diameter” corrections to sun finite angular size are discussed. Finally the section “Summary and conclusion” contains some concluding remarks and a technical offer to practitioners to use spherical mirror as a line-focus collector.

## Spherical mirror

**Figure 1:**
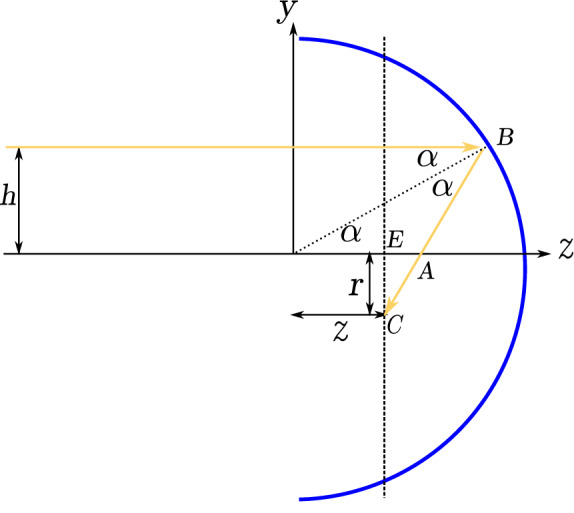
Reflection of a ray from a circular mirror. The Graph is generated using the *Inkscape* software.

In this section, we investigate the concentrated solar irradiation flux profile everywhere, especially in the focal area of a spherical dish of radius *R*, which is exposed to sun beam parallel to its axis. In reality, the rays are not parallel due to the sun disk finite angular size. This point will be considered later but in this section parallel rays are considered. See Fig. 1 that shows the cross-section of the spherical mirror in the *yz* plane, where *z* is the mirror optical axis. With no loss of generality, we take *R* as the unit length and all quantities with length dimension will be scaled with *R*. We set $R=1$ in this paper.

We aim to study the spatial distribution of the light intensity, the concentration of reflected beam, and the region where the light intensity is maximum. This has been done for parabolic trough collectors^26^. For this purpose, we adopt a ray language (geometric optics) in which the light intensity is proportional to the concentration of rays. In this framework, we need to trace each incident ray on the mirror after reflection. Consider an incident ray with lateral distance $h<R$ from the mirror axis onto a spherical mirror of unit radius $R=1$. Let *z* axis be the ray’s direction. The ray hits the mirror at point *B* with the coordinates $y=h$ and $z=\sqrt{1-h^2}$. The ray reflects off the mirror at the point *B* and crosses the *z* axis at point *A*. Denoting the angle of reflection by $\alpha$, and as $OA\, \cos \alpha =\dfrac{R}{2}$, one arrives at:

1 $$\begin{aligned} h=&R\sin \alpha =\sin \alpha , \end{aligned}$$

2 $$\begin{aligned} OA=&\dfrac{R}{2\cos \alpha }=\dfrac{1}{2\cos \alpha }=\frac{1}{2\sqrt{1-h^2}}. \end{aligned}$$

The slope of the reflected line is $\tan 2\alpha$, and the equation of the reflected line is

3 $$\begin{aligned} y-\sin \alpha = (z-\cos \alpha )\tan 2\alpha , \end{aligned}$$

or

4 $$\begin{aligned} y\cos 2\alpha -z\sin 2\alpha +\sin \alpha =0. \end{aligned}$$

**Figure 2:**
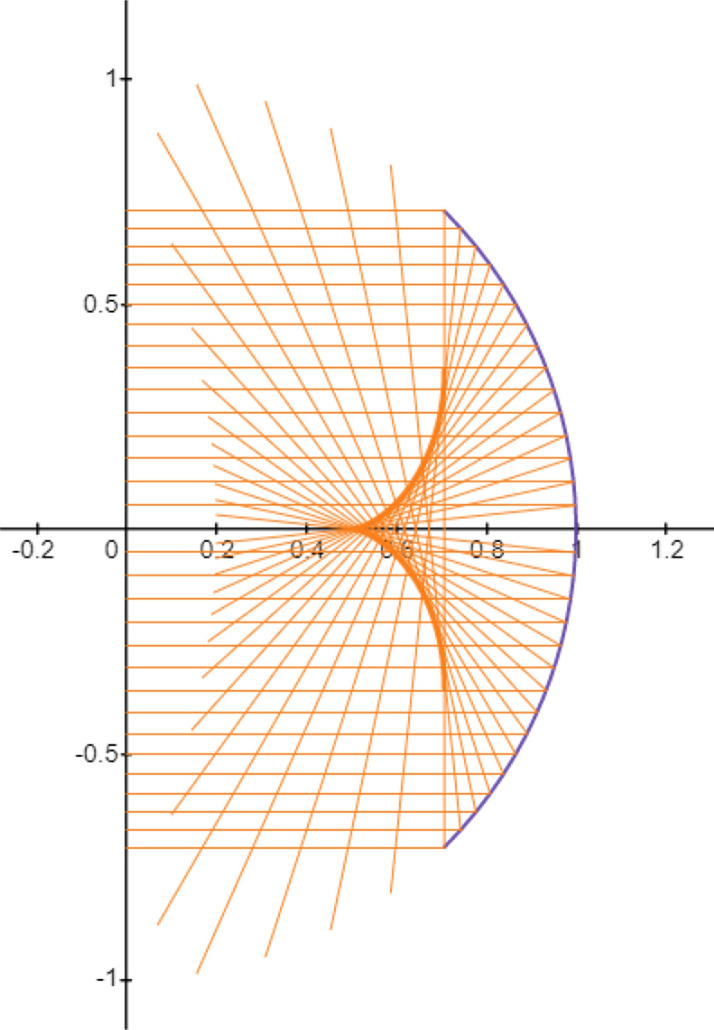
Reflected beam off a spherical mirror (side view). The Graph is generated using the Desmos graphing calculator.

For small values of $\alpha$ (up to a first-order approximation of $\alpha$ in (4)), all the reflected beams pass through the same point (0, 1/2), which may be taken as the quasi focal point of the spherical mirror. In Fig. 2 the incident beam and the corresponding reflected rays are shown.

Some comments are in order: first, for $0 \le h \le \frac{\sqrt{2}}{2}$, i.e.; $\alpha \le \frac{\pi }{4}$, all the reflected rays will hit the optical axis in the interval $[\frac{1}{2},\frac{\sqrt{2}}{2}]$ with positive slope. In fact, for $h=\frac{\sqrt{2}}{2}$ the reflected ray will perpendicularly crosses the *z* axis. Second, for $\frac{\sqrt{2}}{2} \le h\le 1$, i.e.; $\frac{\pi }{4}\le \alpha \le \frac{\pi }{2}$, the reflected rays will hit the optical axis with a negative slope. This means that for *h* larger than $\frac{\sqrt{2}}{2}$ the incident ray may suffer more than one reflection before crossing the optical axis and leaving the mirror. More precisely, For $\frac{\sqrt{2}}{2} \le h\le \frac{\sqrt{3}}{2}$, i.e.; $\pi /4\le \alpha \le \pi /3$, the first reflected ray suffers a second reflection from the mirror surface before crossing the optical axis. For $\frac{\sqrt{3}}{2}<h\le 1~(\pi /3\le \alpha \le \pi /2)$, there may be multiple reflections before crossing the *z* axis. In this article, we avoid double and multiple reflections and hence restrict ourselves to $h \le h_m=\frac{\sqrt{2}}{2}$ ($\alpha \le \frac{\pi }{4}$) where $h_m$ is the mirror aperture radius. Coming back to the problem geometry in Fig. 1, the point *C* shows the crossing point of the reflected ray to the plane perpendicular to the *z* axis located at *z*. Moreover, the point *E* shows the intersection point of *z* axis to this plane. In addition, we show the distances between points *E* and *C* by $r=CE$. From the geometry of Fig. 1 we have:

5 $$r = \left| {\left( {\frac{1}{{2\cos \alpha }} - z} \right)\tan 2\alpha } \right|.$$

Using $\sin \alpha =h$, and somewhat trigonometry, *r* can be expressed in terms of *h* and *z* as follows:

6 $$\begin{aligned} r=h\Big \vert \dfrac{\left( 1- 2z\sqrt{1-h^2}\right) }{1-2h^2}\Big \vert . \end{aligned}$$

**Figure 3:**
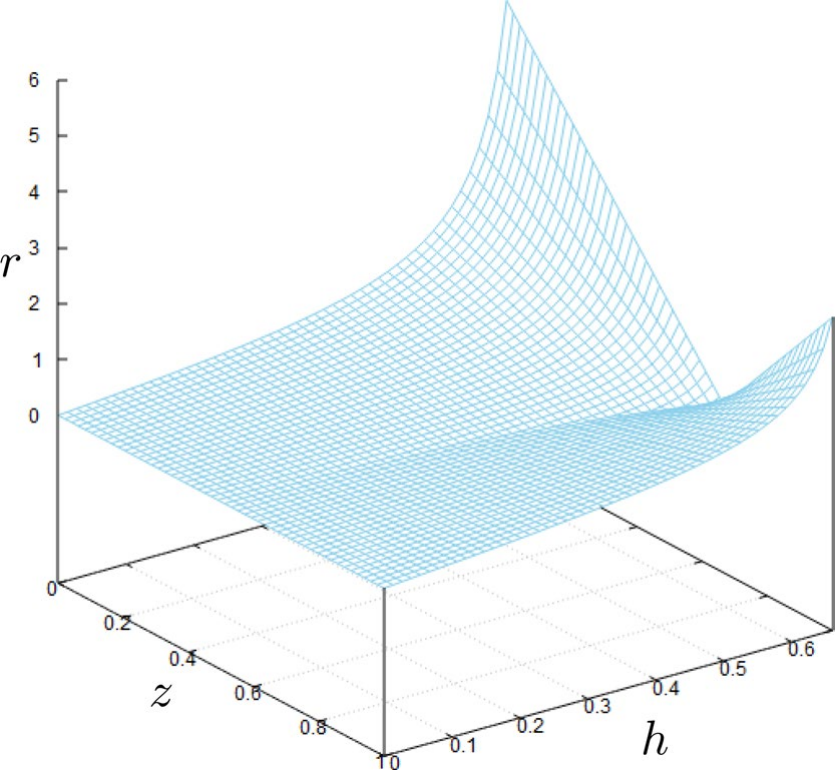
Dependence of *r* on the ray lateral distance *h* from the mirror axis and on the distance *z* of the plane to the origin. The figure has been made with *Gnuplot* 5.4.2 software.

In Fig. 3, *r* is plotted in terms of *h* and *z*.

**Figure 4:**
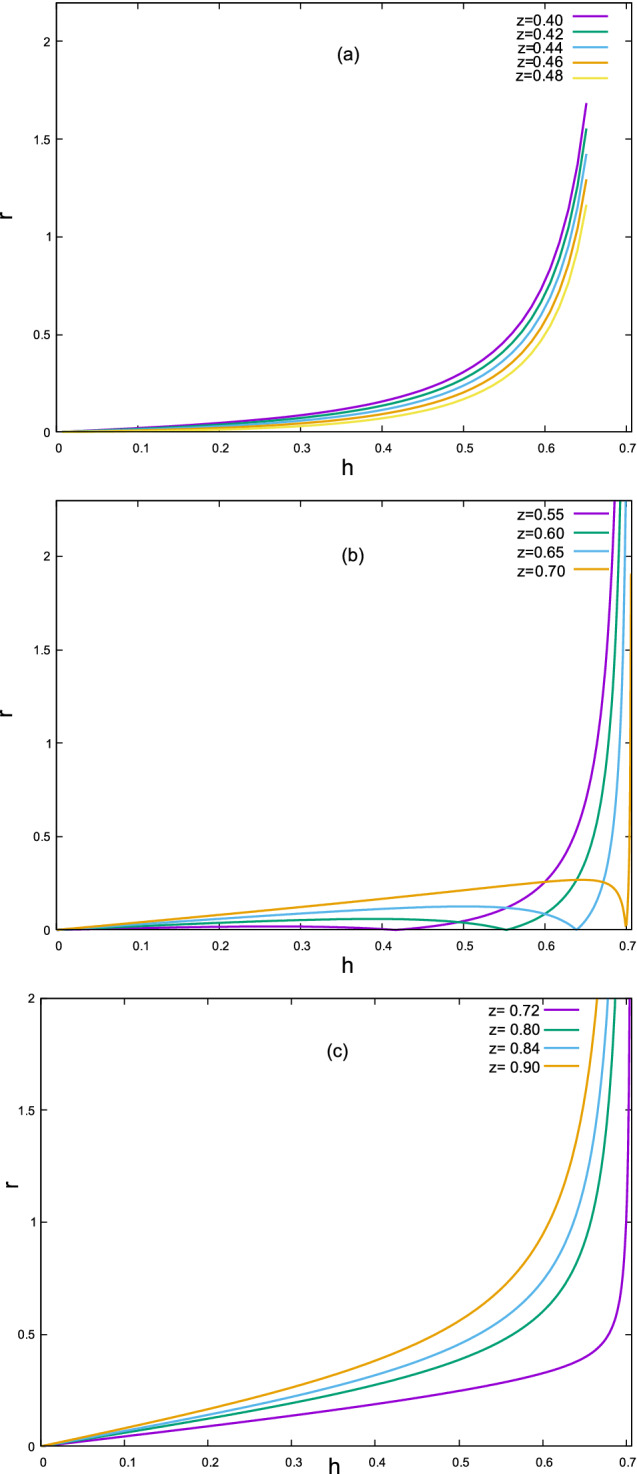
Dependence of *r* on lateral distance *h* from the mirror axis for various values of *z*. The figure has been made with *Gnuplot* 5.4.2 software.

Figure 4 shows the dependence of *r* on *h* for some values of *z*. As it is seen, there is a critical region for *z*. For $0<z< 0.5$ (region I), and $\dfrac{\sqrt{2}}{2}<z<1$ (region III), *r* is a monotonic increasing function of *h*. However, when $0.5<z<\dfrac{\sqrt{2}}{2}$ (region II), there may be one, two or three values of *h* for a given *r* and that there is a maximum value of *r* (except for $h=\sqrt{2}/2$, where *r* diverges). We will soon come back to the physical importance of these regions.

### Flux profile

Now, we wish to study the flux *I* distribution of the reflected beam in the interior of the mirror. The geometry of the problem possesses the axial symmetry, including both the incident beam and also the spherical mirror. Consequently, the reflected beam should also have the same symmetry: the intensity of the reflected beam has no dependence on $\phi$ (azimuth angular variable in the cylindrical coordinates). Due to cylindrical symmetry, radiation intensity *I*(*x*, *y*, *z*), will be a function of *r* and *z* where $r=\sqrt{x^2+y^2}$ is the distance of the intensity observation point to the *z* axis. To find *I* at the observation point (*x*, *y*, *z*), we need to know the lateral distance *h* of the incident ray that after reflection passes the observation point on the constant *z* plane. This is given by Eq.(6) if one succeeds in reversing the function and finds *h* in terms of *r* and *z*. The amount of radiation power intercepted by an annulus of the inner radius *h*, and the outer radius $h+\mathrm{d}h$ on the mirror aperture plane is $2\pi I_0 h\, \mathrm{d}h$, where $I_0$ denotes the incident irradiation intensity. Ignoring any absorption/transmission from the mirror, the radiation power collected by the annulus of the inner radius *r* and the outer radius $r+\mathrm{d}r$ at the constant *z* plane satisfies

7 $$\begin{aligned} 2\pi I_0\, h \mathrm{d}h =2\pi I(r,z)\, r \mathrm{d}r. \end{aligned}$$

Then

8 $$\begin{aligned} \dfrac{I(r,z)}{I_0} =&\Big \vert \dfrac{ h\, \mathrm{d}h}{ r\, \mathrm{d}r}\Big \vert . \end{aligned}$$

where the absolute value is used to ensure the positivity of intensity. To find *I*(*r*, *z*) one requires to know *h*(*r*). Unfortunately, this is not simple. In fact, (6) gives *r* as a function of *h*. Our attempt to find the inverse function *h*(*r*) leads to a quartic equation which is cumbersome to handle:

9 $$\begin{aligned} 4(z^2+r^2)h^4-4rh^3+(1-4r^2-4z^2)h^2+2rh+r^2=0. \end{aligned}$$

As we noticed from Fig. 4, region II needs more care. In regions I ($z\le 0.5$) and III ($z>\dfrac{\sqrt{2}}{2}$) the dependence of *r* on *h* is a one-to-one increasing function. However, in region II ($0.5<z<\dfrac{\sqrt{2}}{2}$) the dependence of *r* on *h* is not one-to-one anymore. More specifically, there is a local maximum $r_m$ and for $r<r_m$ there are three values of *h* which all correspond to a given *r*. See Fig. 4 for clarification. If $r>r_m$ the dependence of *r* on *h* becomes one-to-one again. For the special case $r=r_m$ there are two values of *h* corresponding to $r_m$. Physically speaking, in region II and when $r<r_m$ there are three distinct values of *h* i.e.; $h_1<h_2<h_3$. The associated rays to these triple values of *h* after reflection from the mirror will intersect the plane located at *z* at the same radial distance *r*. As a consequence, the intensity formula should be modified in region II as follows:

10 $$\begin{aligned} I(r,z)=\frac{I_0}{r}\left[ h_1\Big \vert \frac{dh}{dr}\Big \vert _1+h_2\Big \vert \frac{dh}{dr}\Big \vert _2+h_3\Big \vert \frac{dh}{dr}\Big \vert _3\right] . \end{aligned}$$

Let us discuss the singularities of intensity *I*(*r*, *z*). For regions I (Fig. 4a) and III (Fig. 4c) *r*(*h*) is a one-to-one function and we have:

$$\begin{aligned} I(r,z)=\frac{I_0}{r}h\Big \vert \frac{dh}{dr}\Big \vert =\frac{I_0h}{r\Big \vert \dfrac{dr}{dh}\Big \vert }. \end{aligned}$$

**Figure 5:**
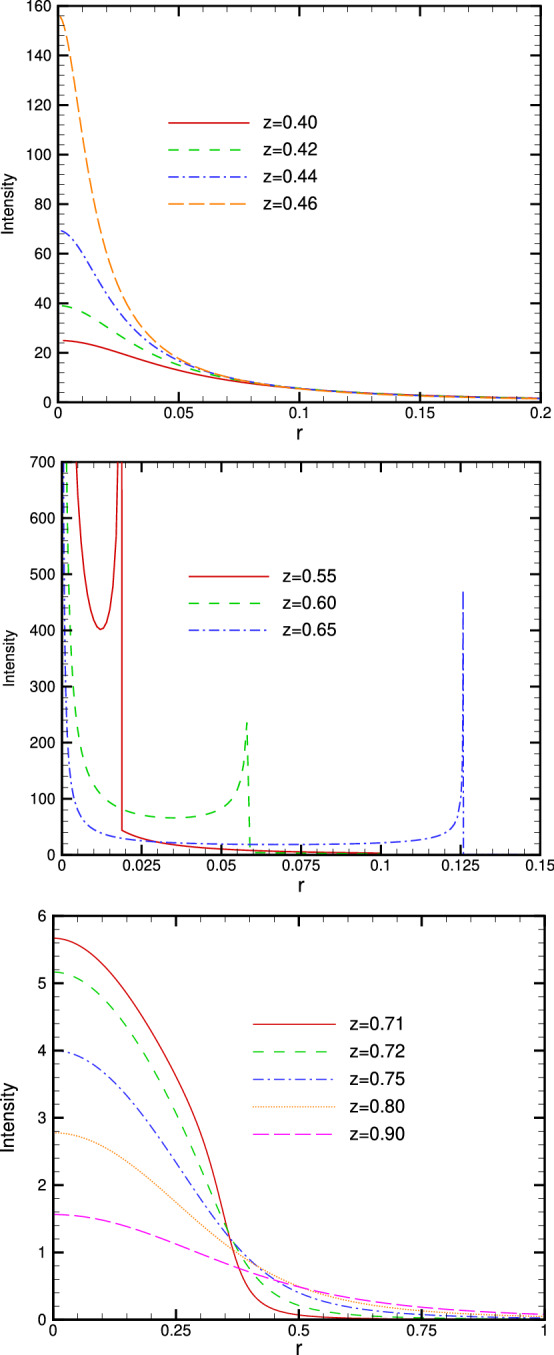
Radial dependence of intensity on the receiver plane at constant *z* for various values of $0<z<0.5$ (top); $0.5<z<\dfrac{\sqrt{2}}{2}$ (middle) and $\dfrac{\sqrt{2}}{2}<z<1$ (bottom). The figure has been made with *Tecplot* 8.0.

Evidently $r=0$ and the solution(s) of $\dfrac{dr}{dh}=0$ are potential values of *r* where the intensity *I*(*r*, *z*) may exhibit a singular behaviour. According to Fig. 4a,c $h \rightarrow 0$ when $r \rightarrow 0$ therefore $r=0$ is not a singularity. To find out the other(s) singularities we should find the solution(s) of $\dfrac{dr}{dh}=0$. However, from Fig. 4a,c we simply find out (graphically) that there is no point where the slope $\dfrac{dr}{dh}$ becomes zero hence there is no singular point *r* in *I*(*r*, *z*). Nevertheless, the situation differs drastically in region II. When $r<r_m$ the point $r=0$ exhibits a singularity in the terms $\dfrac{I_0}{r}h_2\Big \vert \dfrac{dh}{dr}\Big \vert _2$ and $\dfrac{I_0}{r}h_3\Big \vert \dfrac{dh}{dr}\Big \vert _3$ when $r \rightarrow 0$. In fact in the limit $r \rightarrow 0$ we have $h_1(0)=0$ but $h_2(0)=h_3(0)=\dfrac{\sqrt{4z^2-1}}{2z}$. Besides $r=0$, there is a second singularity at $r=r_m$ where $\dfrac{dr}{dh}=0$. The value of $r_m$ can be found via differentiation from Eq. (6). Figure 5 (obtained via a numerical scheme) depicts the radial dependence of intensity in three regions.

**Figure 6:**
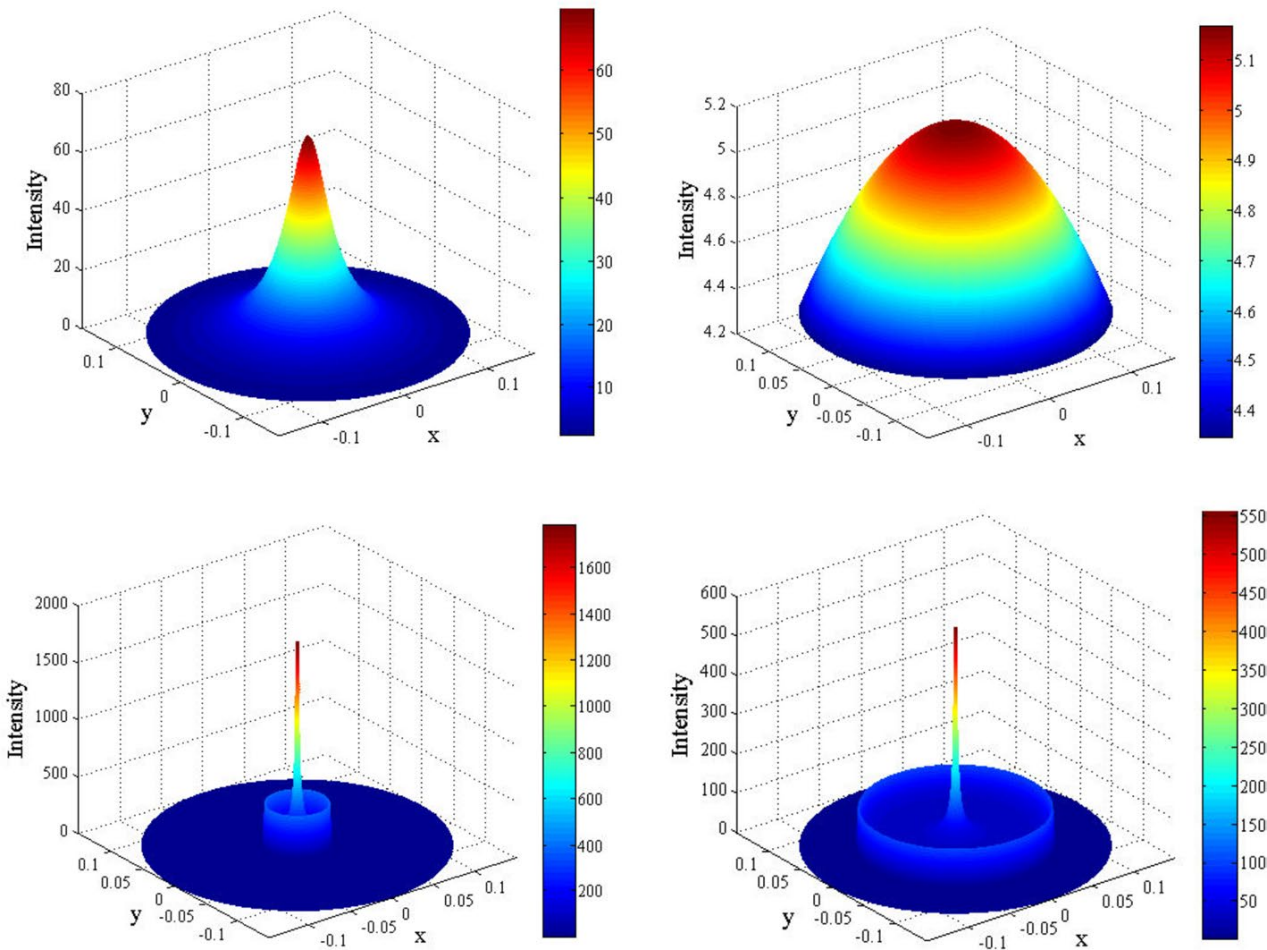
Three dimensional intensity plots on *z* constant surface (colour online). Top left: $z=0.44$ (region I); top right: $z=0.72$ (region III), bottom left: $z=0.57$ (region II) and bottom right: $z=0.63$ (region II). The secondary maxima in region II are noticeable. The figure has been made with *MATLAB* 2009a software.

Figure 6 provides a further insight into the nature of flux concentration in three interior regions of the spherical mirror. The secondary maxima in region II are clearly exhibited.

### envelope of reflected rays

To gain a deeper insight into the nature of flux profile we give a geometrical explanation of intensity profile based on the rays envelope. This is technically known as the so-called *caustic* surface. See e.g.^27^ and the references therein. Let us first define an envelope curve to a set of family curves defined by the continuous parameter parameter $\alpha$.

11 $$\begin{aligned} F(z,y,\alpha )=0, \end{aligned}$$

There may exist a curve such that it is tangent to this family of curves at different points. Tangential points denoted by $(z_t,y_t)$ construct a curve which is called the envelope of the family of curves $F(z,y,\alpha )=0$. The tangential points of two curves of the family which are only infinitesimally different, i.e.; $F(z,y,\alpha )=0$, and $F(z,y,\alpha +\delta \alpha )=0$ are on an infinitesimal intersection of the envelope. In the limit $\delta \alpha \rightarrow 0$ we have:

12 $$\begin{aligned}&F(z_t,y_t,\alpha )=0, \end{aligned}$$

13 $$\begin{aligned}&\dfrac{\partial F(z_t,y_t,\alpha )}{\partial \alpha }=0. \end{aligned}$$

In our problem we take parameter $\alpha$ as the incident angle of a ray. Therefore, each point of envelope of the reflected beam should satisfy

14 $$y_{t} \cos 2\alpha - z_{t} \sin 2\alpha + \sin \alpha = 0,$$

15 $$y_{t} \sin 2\alpha + z_{t} \cos 2\alpha - \frac{1}{2}\cos \alpha = 0.$$

These set of equations leads to the following parametric equations for the envelope:

16 $$y_{t} = \sin ^{3} \alpha ,$$

17 $$z_{t} = \frac{3}{2}\cos \alpha - \cos ^{3} \alpha .$$

**Figure 7:**
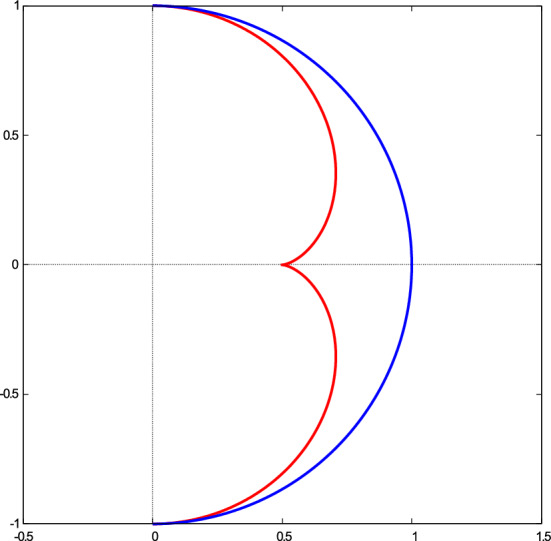
An envelope (red curve) of the reflected beam from the spherical mirror (blue curve). The figure has been made with *Gnuplot* 5.4.2 software.

The envelope has a cusp at the point (0, 1/2), or $\alpha =0$, which is the so-called quasi focal point of the spherical mirror. See Fig. 7 for illustration.

Due to axial symmetry for a spherical mirror, the envelope of reflected rays is a two-dimensional surface having axial symmetry. It is a surface of revolution generated by rotating the curve shown in Fig. 7 around *y* axis.

18 $$r = \sin ^{3} \alpha ,$$

19 $$z = \frac{3}{2}\cos \alpha - \cos ^{3} \alpha .$$

**Figure 8:**
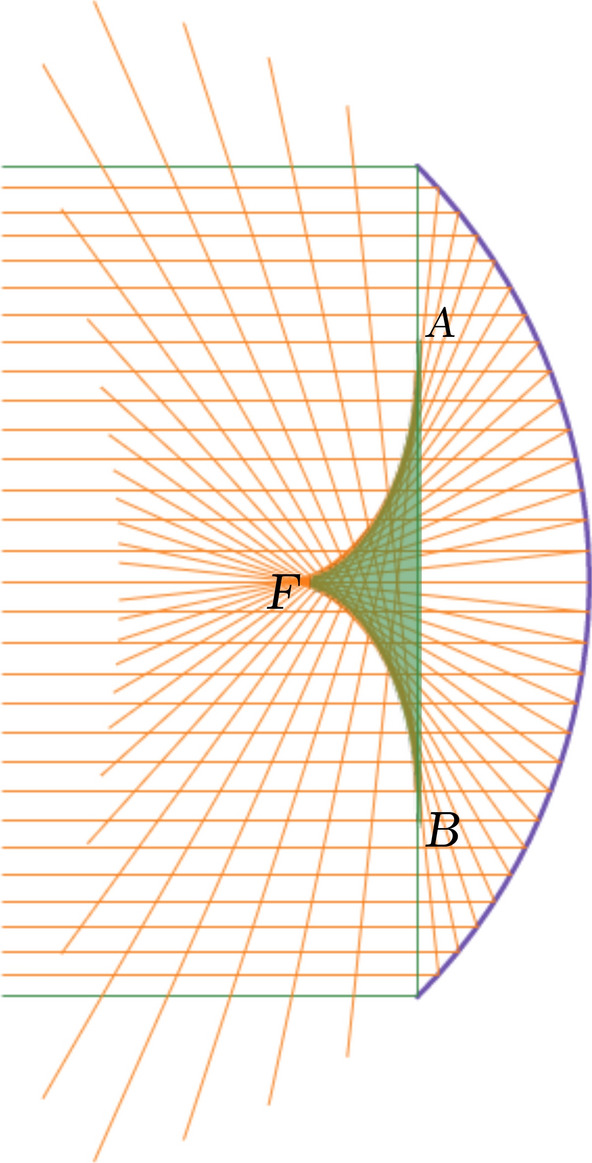
Envelope of the reflected beam and highly concentrated region (region II). The figure has been made with Desmos graphing calculator.

All of the reflected beam will enter the disk, shown by the line *AB* in the Fig. 8, passing through the green region and exit from the revolutionary surface of the envelope shown by the segments *FA*, and *FB* in the figure. The geometry of the green region in Fig. 8 can be of practical importance. In fact, it can give solar thermal engineers a hint how to optimise the design of a cavity receiver. An optimal design could have a horn-like shape in accordance to the highly concentrated green region^12,13,22,23^.

We can express *h* and *r* in terms if $\alpha$ as follows:

20 $$\begin{aligned} h =&\sin \alpha \nonumber \end{aligned}$$

21 $$\begin{aligned}r=&\big \vert (\dfrac{1}{2\cos \alpha }- z)\tan 2\alpha \big \vert=\big \vert \frac{-\sin \alpha (1-2z\cos \alpha )}{2\cos ^2\alpha - 1}\big \vert , \end{aligned}$$

$\alpha =\arccos (\dfrac{1}{2z})$ leads to a non-physical solution for $\alpha$ in the regions I and III. Using (20) and (21) we can evaluate $\dfrac{rI(r,z)}{I_0}$ in terms of parameter $\alpha$:

22 $$\begin{aligned} \dfrac{rI(r,z)}{I_0} =&\dfrac{(2\cos ^2\alpha - 1)^2\sin \alpha \cos \alpha }{2\cos ^3\alpha - 3\cos \alpha + 2z}, \end{aligned}$$

It diverges at

23 $$\begin{aligned} z=-\cos ^3\alpha _m+\frac{3}{2}\cos \alpha _m, \end{aligned}$$

Substitution of this *z* in (21) gives $r_m= \sin ^3 \alpha _m$ the radius for which $r_mI(r_m,z)$ diverges. Therefore, for any $0<z<1$, there is ring of radius $r_m$ on the plane of constant *z*, at which the intensity diverges. Geometrically this radius is the intersection of the constant *z* plane and the envelope.

## Ray tracing Monte Carlo simulation

**Figure 9:**
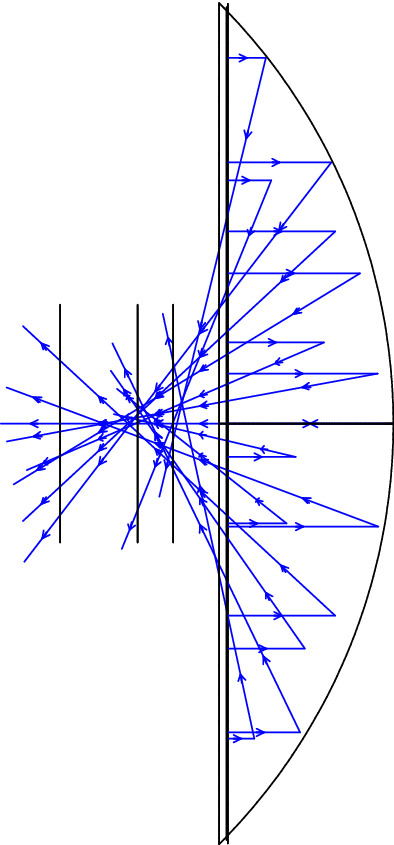
Optical setup of ray tracing in non-sequential mode. The figure has been made with *Zemax* 13 R2 SP4 software.

In order to check the accuracy and validation of our theoretical analysis, we performed Monte Carlo ray tracing simulations. Monte Carlo simulation is a useful tool for characterising the properties of systems constituting a large number of interacting particles and has been applied to many areas of science and engineering during the past decades^28,29^. The Monte-Carlo ray-tracing methodology replicates the real photon interactions, in which stochastic paths of a large number of rays are followed as they interact with surfaces. We have used the Zemax, a widely used commercial optical software, as our optical simulation software. Figure 9 shows the optical setup of ray tracing in non-sequential mode. We use the circle source and the Sobol sampling method with $N=10^7$ rays to model a collimated spread source. All of the rays are emitted from the source parallel to the z-axis. Several rectangular detectors are placed in optical axis in order to the record the intensity distribution. The detectors have 800 × 800 pixels and, 40 × 40 cm^2^ area.

**Figure 10:**
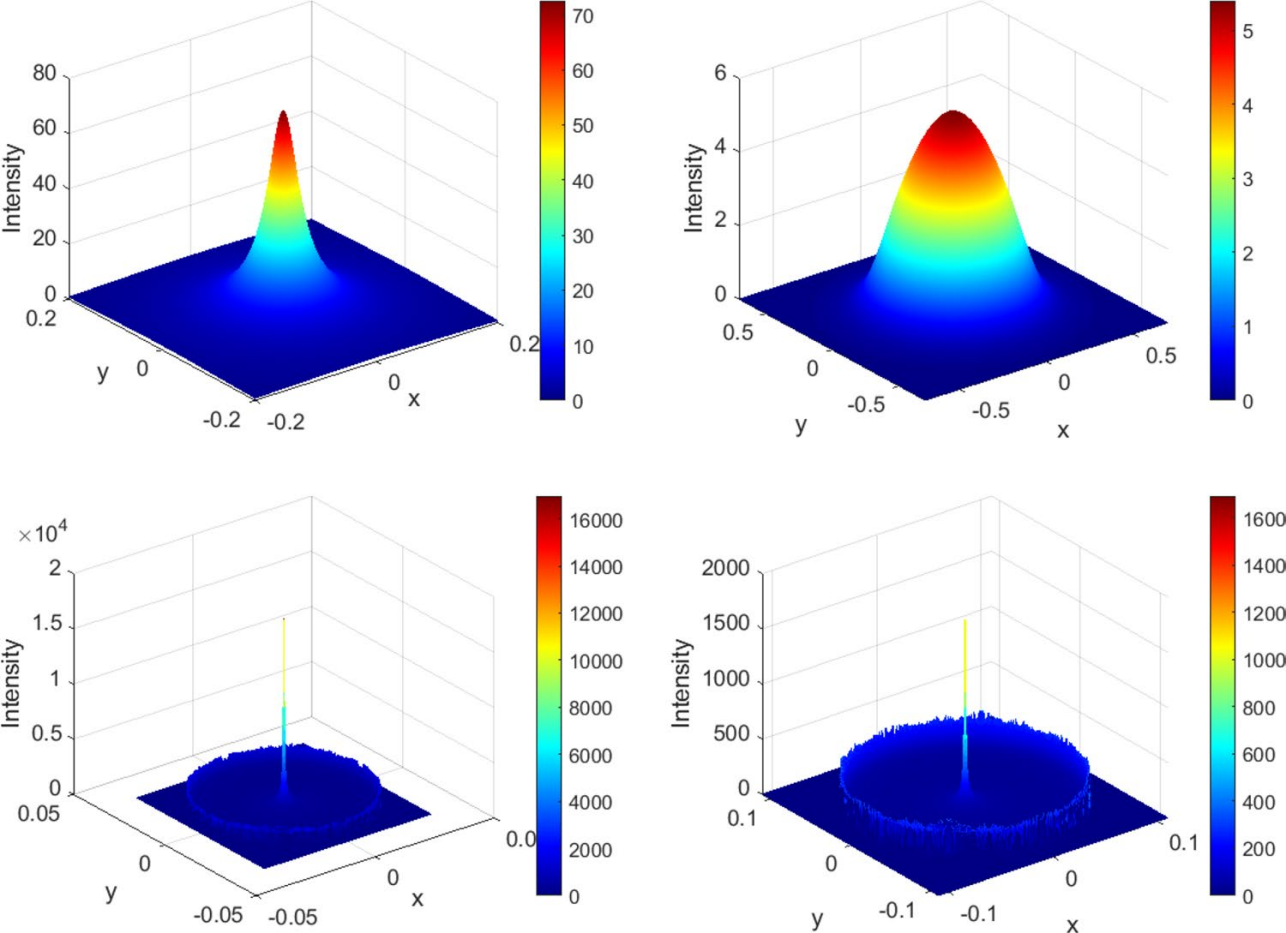
Three dimensional intensity plots, obtained by Monte Carlo ray tracing simulation, on *z* constant surface (colour online). Top left: $z=0.44$ (region I); top right: $z=0.72$ (region III), bottom left: $z=0.57$ (region II) and bottom right: $z=0.63$ (region II). The secondary maxima in region II are noticeable. The figures have been made with *Zemax* 13 R2 SP4 software.

The results of ray tracing Monte Carlo simulations are shown in Fig. 10. The figure includes three dimensional colour plots of intensity profiles at various distances *z* of the receiver plane. As you can see the results of Monte Carlo simulation are in a good agreement with analytical results. The slight difference is due to finite difference approximation of derivatives in (10), finite number of rays, and Monte Carlo systematic errors.

## Experiment and empirical data

**Figure 11:**
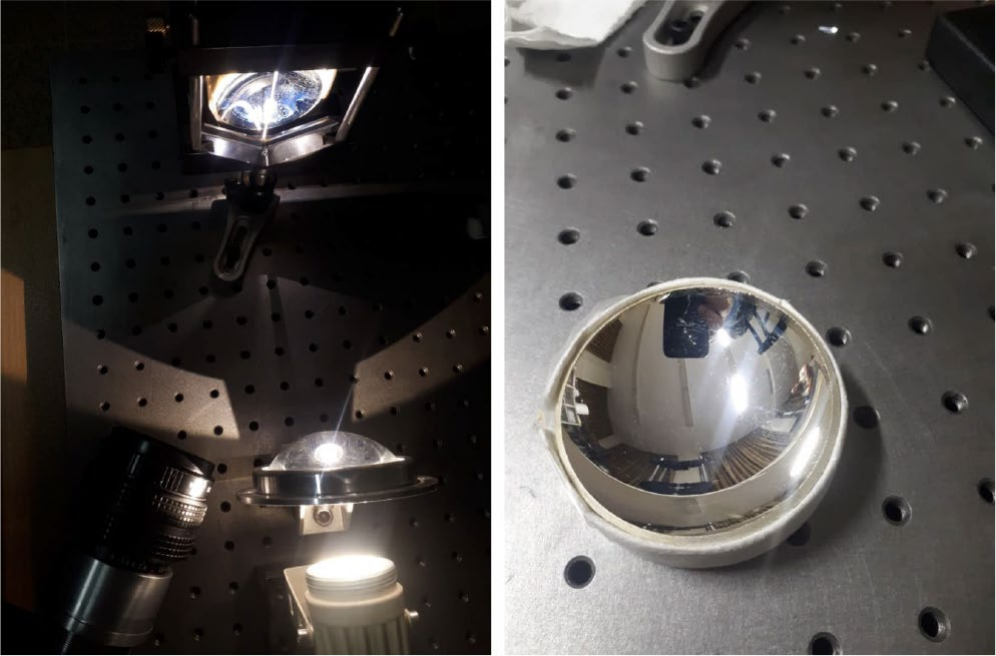
Left: The measurement optical setup. A ribbon of white paper with the size 1.2 × 70 mm^2^ is placed close to focal point. The mirror is translated by the micro positioner along the spherical mirror optical axis. Right: silver coated lens spherical mirror.

**Figure 12:**
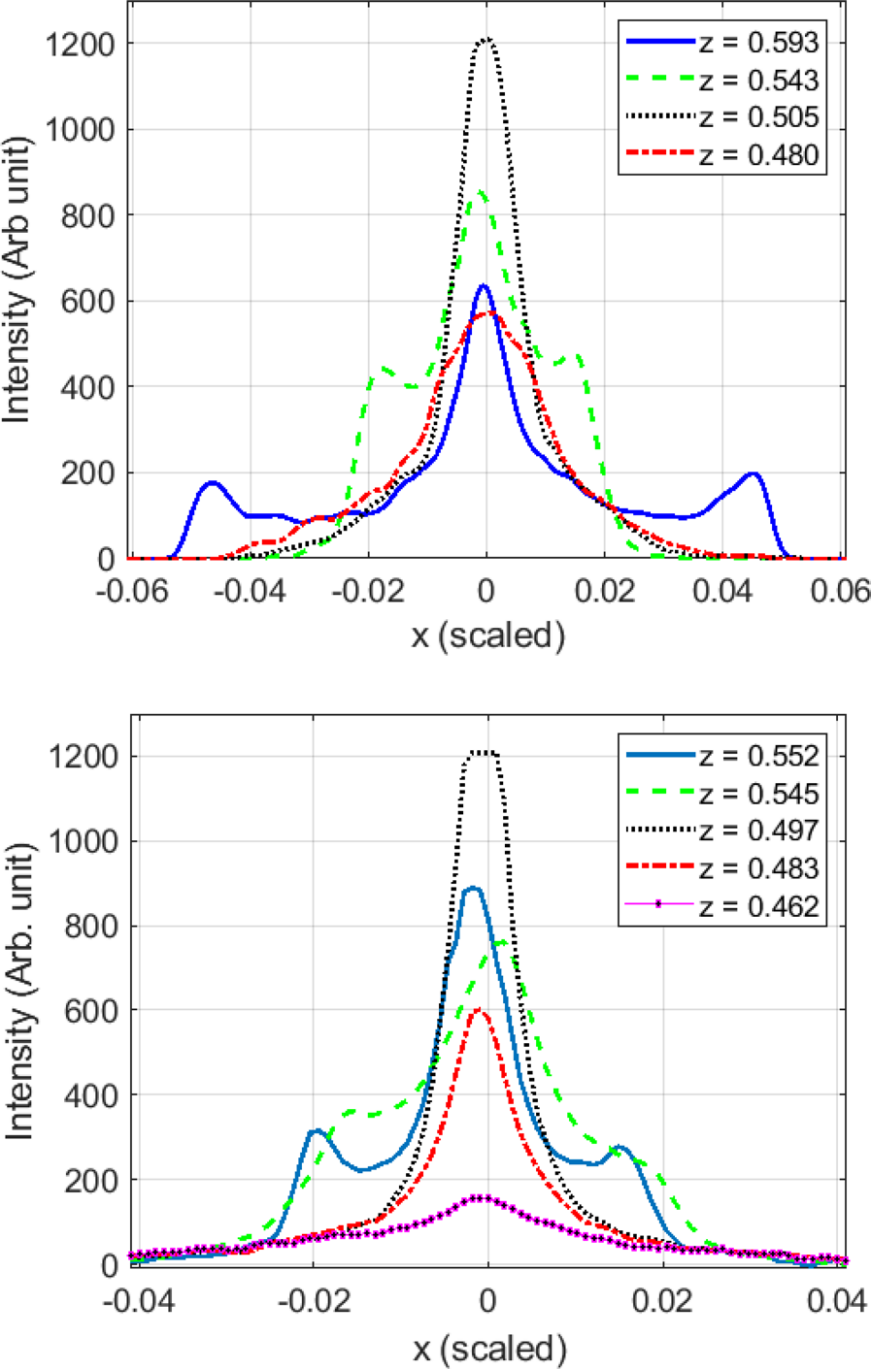
The profile of spot intensity in regions close to focal point $f=\frac{R}{2}$ of the spherical mirror ($R=39.0~$mm). The figures have been made with *MATLAB* 2020a software.

In order to validate our theoretical results, an experiment was devised. In this lab experiment a small spherical mirror was exposed to a parallel beam of light. The spherical concave face of a negative ophthalmic lens is used as the spherical reflector. The ophthalmic lens diopter is $-18$ and, its refractive index is $n=1.7$. This ophthalmic lens is flat-concave. The lens clear aperture is $d=61.5~mm$ and the concave face depth is $h=22.0~mm$. The surface radius of spherical curvature *R* is determined from the equation:$R=(r^2+h^2)/2h$ where *r* is the aperture radius and *h* is the concave face depth. The formula gives:$R =39.0~mm$. The concave face is silver coated with DC sputtering technique. A collimated beam of high bright white LED light is projected onto the spherical surface. The LED source is placed 10 *m* away from the mirror to satisfy the plane wave propagation criteria. The plane wave of light is incident to the mirror and reflected. A narrow ribbon of white paper ($1.2\times 70~mm^2$) which scatters the light is placed near the focal plane. An imaging system was placed in front of the diffuser (white paper ribbon) and acquires the image of the spot profile. The setup is shown in Fig. 11. We use a 10-bits coloured CCD camera to capture the images. An aberration corrected lens with focal distance of 75 mm mapped the image of spot profile into the CCD sensor. The white ribbon was fixed on a mechanical holder and the spherical mirror was placed on a micro positioner stage. The distance *z* between ribbon and the mirror is changed from $z=15$ mm to $z=22$ mm with the $\delta z=25~\mu m$ span. In any step, the intensity profile at the focal point is captured. Figure 12 exhibits the intensity profile for some values of *z*. All distances are scaled with the spherical mirror radius *R*. As you can see, there is a satisfactory overall agreement between empirical data and theoretical findings. Most importantly, the second intensity peak in the region $II (0.5\le z \le 0.71)$ is observed in experiment. However, the second peak is smoothed and not so sharp as the theoretical results. The reasons are mainly due to imperfection and deviation of reflector surface from purely theoretical one, and partial ability of the collimator to produce a parallel beam of light. Note that in experiment there is no more a radial symmetry. We actually measured the line profile along an arbitrary direction. The empirical results for other directions were similar in nature to the one presented in the paper.

## Concentration ratio

In the previous section we explored the spatial dependence of the radiation flux in the focal area of a spherical mirror. As we noticed, analogous to a parabolic mirror, a spherical mirror is also able to concentrate the solar irradiation. However, the nature of concentration is substantially different. Particularly, there is no focal point. Nevertheless, we may consider the point *R*/2 as the quasi focal point of the mirror because the intensity is notably large in the vicinity of $z=R/2$. In this section we discuss the receipt of concentrated irradiation near quasi focal area and its physical aspects. To receive the concentrated light, we place a receiver in the focal area where intensity is large. Normally the receiver is placed vertically to the optical axis. The main question is where should the receiver be placed to maximize the amount of irradiated flux on it? To address this question the concept of concentration ratio has been introduced in the literature^6,30^. Principally two types of concentration ratio is defined: geometric and radiation. Geometric concentration ratio is defined as the ratio of the dish aperture area to the area of the focal spot. The ambiguity arises when we want to define the focal spot size. The radiation-based concentration ratio is defined as the averaged intensity over the flat receiver area, divided by the incident beam intensity onto the collector aperture. Here we obtain the radiation-based concentration ratio for a flat circular receiver of dimensionless radius *a*. The circular receiver is placed perpendicular to the *z* axis at distance *z* from the mirror origin. The receiver centre is on the *z* axis. By definition, the concentration ratio is:

24 $$\begin{aligned} C(a,z):=&\dfrac{1}{I_0}\dfrac{\int _0^aI(r,z)\, 2\pi r \mathrm{d}r}{\pi a^2 }. \end{aligned}$$

As it is seen in Fig. 4, for the regions I ($0<z<0.5$), and III ($\sqrt{2}/2<z<1$), there are one to one correspondence between *h* and *r*, and we can simply evaluated the integral in (24). Replacing $rI(r,z)\mathrm{d}r$ from (7) by $h\mathrm{d}h$ in the integrand of (24) it turns out:

25 $$\begin{aligned} C(a,z)=\dfrac{h^2(a,z)}{a^2}, \end{aligned}$$

where *h*(*a*, *z*) is the solution of (6) with $r=a$. Physically speaking, all the rays with lateral distance smaller than *h*(*a*, *z*) will hit the receiver therefore the amount of irradiation intercepted by the receiver will be $I_0$ times the area of the circle with radius *h*(*a*, *z*) in the aperture plane. In region II ($0.5<z<\sqrt{2}/2$), one needs to be cautious. Regarding the triple solutions for *h* in this region we have:

26 $$\begin{aligned} \int _0^aI(r,z)\, r \mathrm{d}r=&\int _0^{h_1}h\, \mathrm{d}h + \int _{h_2}^{h_3}h\, \mathrm{d}h\nonumber \\ =&h_1(a,z)^2-h_2(a,z)^2+h_3(a,z)^2. \end{aligned}$$

**Figure 13:**
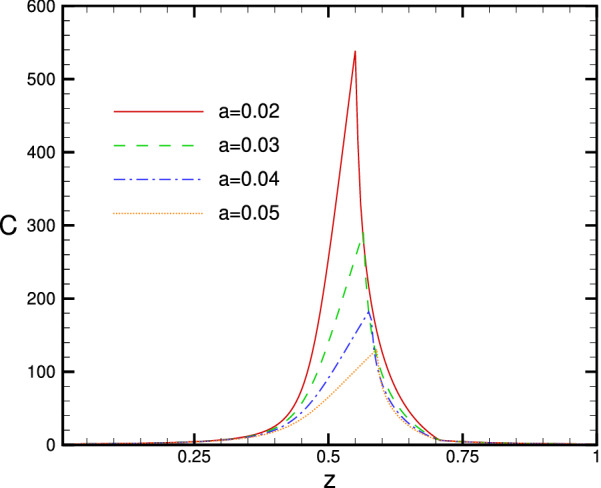
Plot of *C*(*a*, *z*) in terms of *z* for some values of receiver radius *a*. The figure has been made with *Tecplot* 8.0.

Physically speaking none of the rays with lateral distance between $h_1(a,z)$ and $h_2(a,z)$ will hit the receiver disk. Those which hit the disk come from two regions: $0\le h \le h_1(a,z)$ and $h_2(a,z)\le h \le h_3(a,z)$. See Fig. 4b for illustration. The concentration ratio will therefore be:

27 $$\begin{aligned} C(a,z)=\dfrac{1}{a^2}[h_1(a,z)^2-h_2(a,z)^2+h_3(a,z)^2], \end{aligned}$$

where $h_i$’s are the solutions of (6) ($h_1\le h_2\le h_3$) which can be found numerically. Figure 13 depicts the dependence of the concentration ratio on the receiver distance *z* from the mirror centre for a circular receiver disk with reduced radius *a*.

### Line receiver

As it is seen from Fig. 8 all the reflected rays passing through the aperture with $h<h_\mathrm{max}=\sqrt{2}/2$, cross the *z* line in the range $0.5\le z\le \sqrt{2}/2$. This implies that any line receiver on this range could collect all the incident beam. Here we wish to obtain the power per unit length by a line receiver, denoted by *J*(*z*) on the *z* axis. Conservation of irradiated power gives:

28 $$\begin{aligned} J(z)\mathrm{d}z =2\pi I_0 h\mathrm{d}h. \end{aligned}$$

which gives

29 $$\begin{aligned} J(z)=2\pi I_0 h\dfrac{\mathrm{d}h}{\mathrm{d}z} ={\left\{ \begin{array}{ll} \dfrac{\pi I_0}{2z^3}&{} 0.5\le z\le \sqrt{2}/2\\ 0&{}\mathrm{elsewhere} \end{array}\right. } \end{aligned}$$

**Figure 14:**
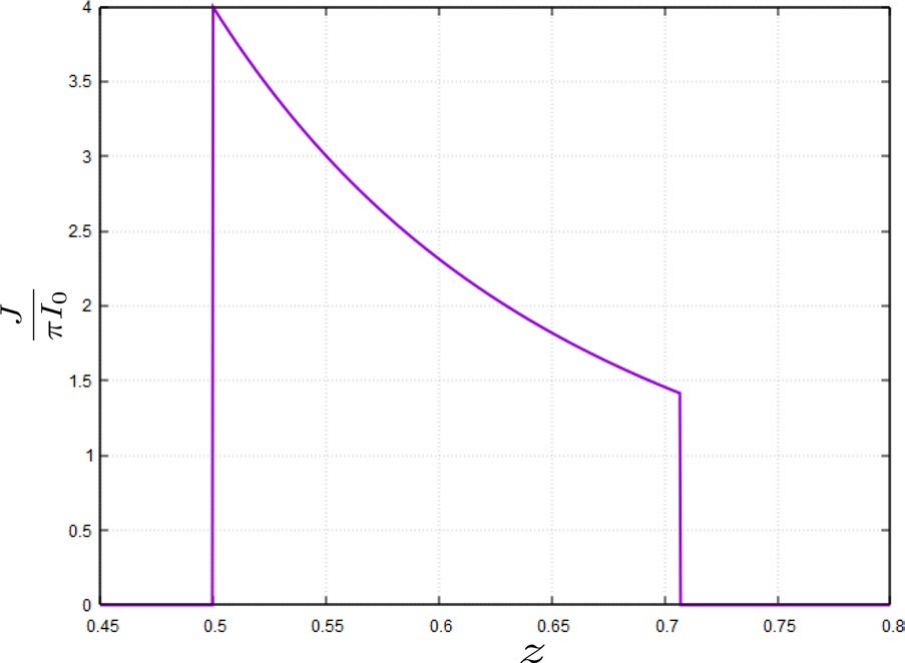
$\dfrac{J(z)}{\pi I_0}$ in terms of *z* for a line receiver on the *z* axis. The figure has been made with *Gnuplot* 5.4.2 software.

**Figure 15:**
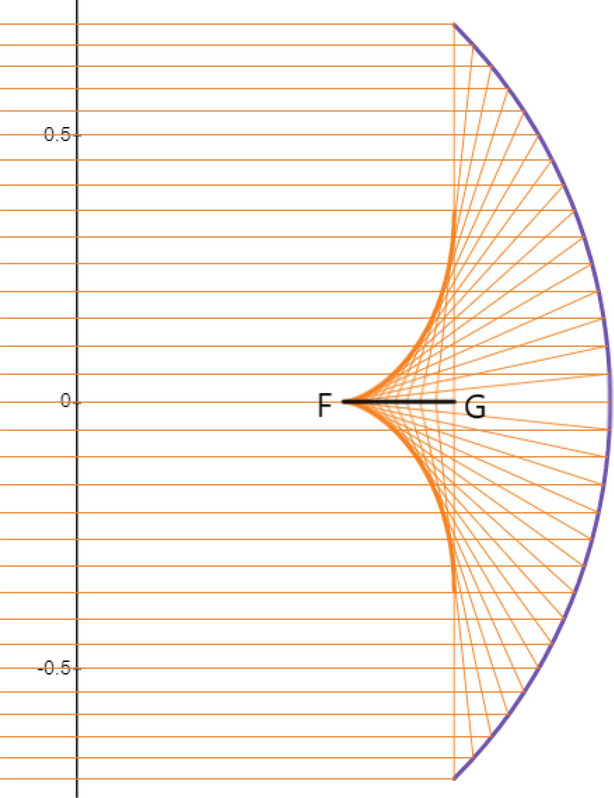
Line radiation concentration on the optical axis of a spherical mirror. All the reflected radiation is concentrated on the line segment FG having the length $l=\frac{\sqrt{2}-1}{2}$. The Graph is generated using the Desmos graphing calculator.

We recall that the reflected ray which intersects the *z* axis at *z* has originated from the incident ray having the lateral distance *h* where $z=\frac{1}{2\sqrt{1-h^2}}$. We can simply reverse the formula and obtain *h* as a function of *z* as follows: $h=\frac{\sqrt{4z^2-1}}{2z}$. This allows us to obtain $\frac{dh}{dz}$ needed in Eq. (29). Figure 14 shows the dependence of line flux *J*(*z*) on *z*:

In contrast to a paraboloidal dish which concentrates the incoming radiation at a focal point, a spherical mirror concentrates the incoming irradiation on a piece of line on the optical axis. The line starts from $R/2=1/2$ but its endpoint depends on the mirror aperture radius. In this paper with $h_m=\frac{\sqrt{2}}{2}$ the line ends at $z=\frac{\sqrt{2}}{2}$. See Fig. 15 for illustration.

The fact that all the reflected power is concentrated on the mirror axis stimulates the idea of a line-focus type receiver. Despite a parabolic trough is the typical solar line-focus concentrator^2,4^ we have managed to show that a spherical mirror can also act as a novel candidate of line-focus solar collector. More technically, a spherical mirror can concentrate the irradiation both in a quasi focal area and on a line segment. To explore the degree of axial concentration let us consider a rectangular region in the $x-z$ plane. This region has a width *w* and length $l=\dfrac{\sqrt{2}-1}{2}=0.205$ (in reduced unit) with its symmetry axis along the *z* axis. All the reflected beam crosses this rectangular region. The concentration ratio of the receiver is simply the ratio of the aperture area to the rectangular region area:

30 $$\begin{aligned} C(w,l)=\dfrac{\pi /2}{lw}=\dfrac{\pi }{w(\sqrt{2} -1)}\approx \dfrac{7.66}{w}. \end{aligned}$$

Theoretically, the concentration ration diverges as $w\rightarrow 0$, but there are practical limitations on the receiver width. To estimate the typical value of *w* let us consider a vacuum tube receiver that is used in line-focusing systems such as linear troughs and linear Fresnel collectors^6^. The typical diameter of the tube is about 5 centimeters. Taking $R=5~m$ as a typical radius for solar concentrator spherical mirror one finds the value of $w=\frac{0.05}{5}=0.01$ in reduced unit. It turns out that $C(0.01,0.205)=\dfrac{7.6}{w}=760$ which is even larger than the concentration ratio of the disk receiver.

## Comparison to parabolic dish

In this section we compare the spherical mirror degree of concentration to its counterpart in a paraboloidal one. Consider a paraboloidal dish of focal length *f* and equivalent circular aperture area to that of the spherical mirror that is $A=\pi h_m^2$ with $h_m=\dfrac{\sqrt{2}}{2}$. Analogous to the spherical mirror we work in a dimensionless unit where the focal length, $f=1$, is set as the unit length. Mathematically it is known that all the incident rays parallel to the paraboloidal mirror axis will cross the focal point upon reflection from the mirror. On the other hand, the radiation intensity has an axial symmetry like the spherical mirror and hence $I_{pb}=I_{pb}(r,z)$. Point concentration gives $I_{pb}(r,z=f) \sim \delta (r)$. This means that theoretically, the concentration ratio (geometric or radiation) can be indefinitely large. However, practical restrictions and the sun disk finite angular diametre (0.009 radian)^31^ prevent to have an infinite concentration ratio. Let us obtain the concentration ratio of a paraboloidal dish. Consider a circular receiver of radius *a* which is perpendicular to the dish optical axis and located at $z=f$. The receiver absorbs all the intercepted solar power that is $I_0$ times the dish aperture area. The average flux on this receiver turns out to be:

31 $$\begin{aligned} {\bar{I}}_{pb}(a)=\dfrac{I_0\pi /2}{\pi a^2}. \end{aligned}$$

The corresponding concentration ratio turns out to be:

32 $$\begin{aligned} C_{pb}(a)={\bar{I}}_{pb}(a)/I_0=\dfrac{1}{2a^2}. \end{aligned}$$

as an estimation of the receiver aperture size we put $a=0.02~(10~cm)$^13,32^ and find $C_{pb}(0.02)=1250$, which is of course higher than the maximal concentration ratio of the spherical mirror having the same aperture area. Despite theoretically the concentration ratio of a paraboloidal dish is higher than a spherical one but we should notice that this might not be valid when practical limitations are taken into account. First, the size of cavity receivers which are used in point-focus concentrators cannot be arbitrarily small. Second, the heat loss from cavity receivers is higher than the vacuum tube receivers which are used in line-focus systems. Therefore, it is not known a priori which receiver type can exhibit a better performance. To find which type of dish has a higher efficiency, we offer an experimental study which can compare the thermo-optical performances of a paraboloidal dish with a cavity receiver and a spherical dish (having identical aperture area) with a vacuum tube receiver.

## Sun angular diameter

**Figure 16:**
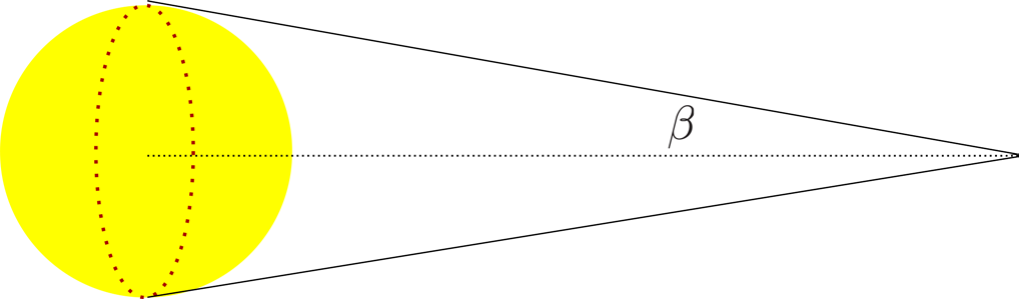
Sun is an extended light source whose angular diameter is $2\beta \approx 0.53^\circ$. The Graph is generated using the *Inkscape* software.

When the angular size of a light source is less than the instrument resolution used to observe it, one can assume the light source is a point source. Otherwise, we should take it as an extended source. Let us consider the more realistic case, where the sun is not a point source but an extended one. Angular diameter or size of the sun seen from the Earth is $2\beta \approx 0.53^\circ$, See Fig. 16. Consequently, incident rays on Earth are not parallel, but are up to about half a degree out of parallel.

**Figure 17:**
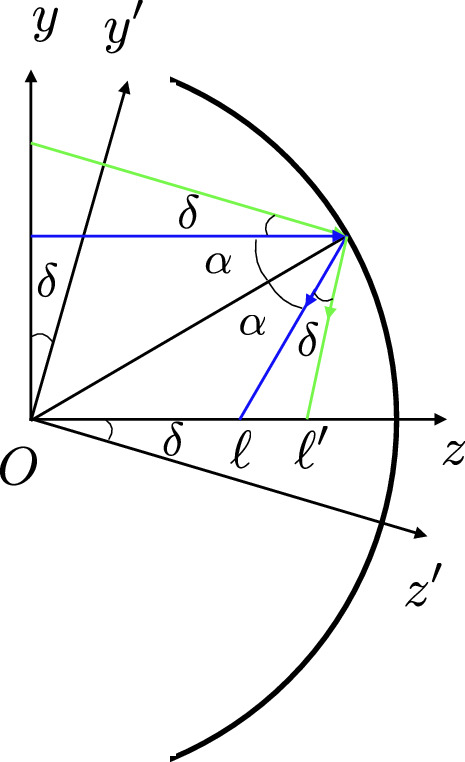
While the incident angle of a light ray is $\delta$, it is easier to solve the problem in a reference frame, rotated by an angle $\delta$. The Graph is generated using the Keynote.

While the incident angle of a light ray is $\delta$, it is easier to solve the problem in the reference frame, $y'-z'$, which is rotated rotated by an angle $\delta$ with respect to the $y-z$ frame. See Fig. 17 for details. In the rotated frame, each point of the reflected beam envelope should satisfy

33 $$y_{t}^{\prime } \cos 2(\alpha + \delta ) - z_{t} \sin 2(\alpha + \delta ) + \sin (\alpha + \delta ) = 0,$$

34 $$y_{t}^{\prime } \sin 2(\alpha + \delta ) + z_{t} \cos 2(\alpha + \delta ) - \frac{1}{2}\cos (\alpha + \delta ) = 0.$$

This set of equations leads to the following parametric equations for the envelope:

35 $$y_{t}^{\prime } = \frac{3}{4}\sin 3(\alpha + \delta ) - \frac{1}{4}\sin 3(\alpha + \delta ),$$

36 $$z_{t}^{\prime } = \frac{3}{4}\cos 3(\alpha + \delta ) - \frac{1}{4}\cos 3(\alpha + \delta ).$$

The envelope in $y-z$ frame is:

37 $$y_{t} = \frac{3}{4}\sin \alpha - \frac{1}{4}\sin (3\alpha + 2\delta ),$$

38 $$z_{t} = \frac{3}{4}\cos \alpha - \frac{1}{4}\cos (3\alpha + 2\delta ).$$

Due to axial symmetry of a spherical mirror, the envelope of reflected rays is a two-dimensional surface with axial symmetry. Indeed, it is a surface of revolution generated by rotating the curve given by equations (37,38) around *y* axis.

39 $$r = \left| {\frac{3}{4}\sin \alpha - \frac{1}{4}\sin \left( {3\alpha + 2\delta } \right)} \right|,$$

40 $$z = \frac{3}{4}\cos \alpha - \frac{1}{4}\cos (3\alpha + 2\delta ).$$

The cusp will appear when *z* takes its minimum value.

41 $$3\sin \alpha _{m} - \sin (3\alpha _{m} + 2\delta ) = 0.$$

Note that for small values of $\delta$, $\alpha _m\approx -\delta$. Up to first order of $\delta$ the cusp position is:

42 $$\begin{aligned}&r_m=|\dfrac{\delta }{2}|, \end{aligned}$$

43 $$\begin{aligned}&z_m=\dfrac{1}{2}. \end{aligned}$$

**Figure 18:**
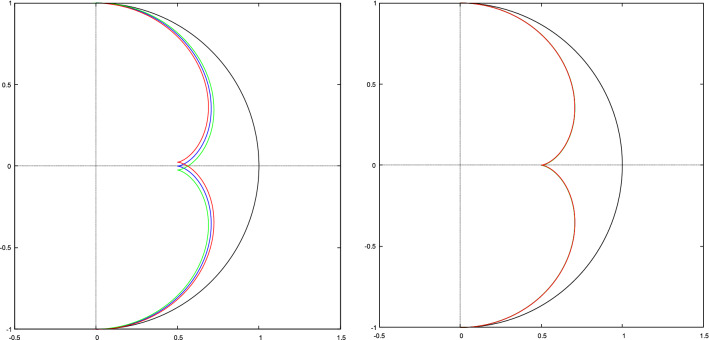
As the sun is an extended light source, the cusp will be broadened. The boundaries of the broadened region are red and green curves. In the right figure, $\beta$ is taken to be its real value. For a dish of radius $5\, \mathrm{m}$, the broadened cusp is a spot of radius $r_s\approx 1.1\, \mathrm{cm}$. In the left figure, $\beta$ is taken to be much larger to show the effect of sun diameter on the cusp broadening. The figure has been made with *Gnuplot* 5.4.2 software.

Because the incident rays are non-parallel, the incident angle may deviate from parallel rays by $-\beta \le \delta \le \beta$. Consequently, the cusp will be broadened. The boundaries are shown by red and green lines in Fig. 18. In the right figure, $\beta$ is taken its real value which is about $0.26^\circ$, but in the left figure, $\beta$ is exaggeratedly taken much larger to show the effect of sun diameter on the cusp broadening. The cusp is not a point anymore and it is a spot of radius $r_s\approx \dfrac{\beta }{2}$. We recall that the mirror radius has been set as the unit length. For a dish of radius $5\, \mathrm{m}$, it is $r_s\approx 1.1\, \mathrm{cm}$.

**Figure 19:**
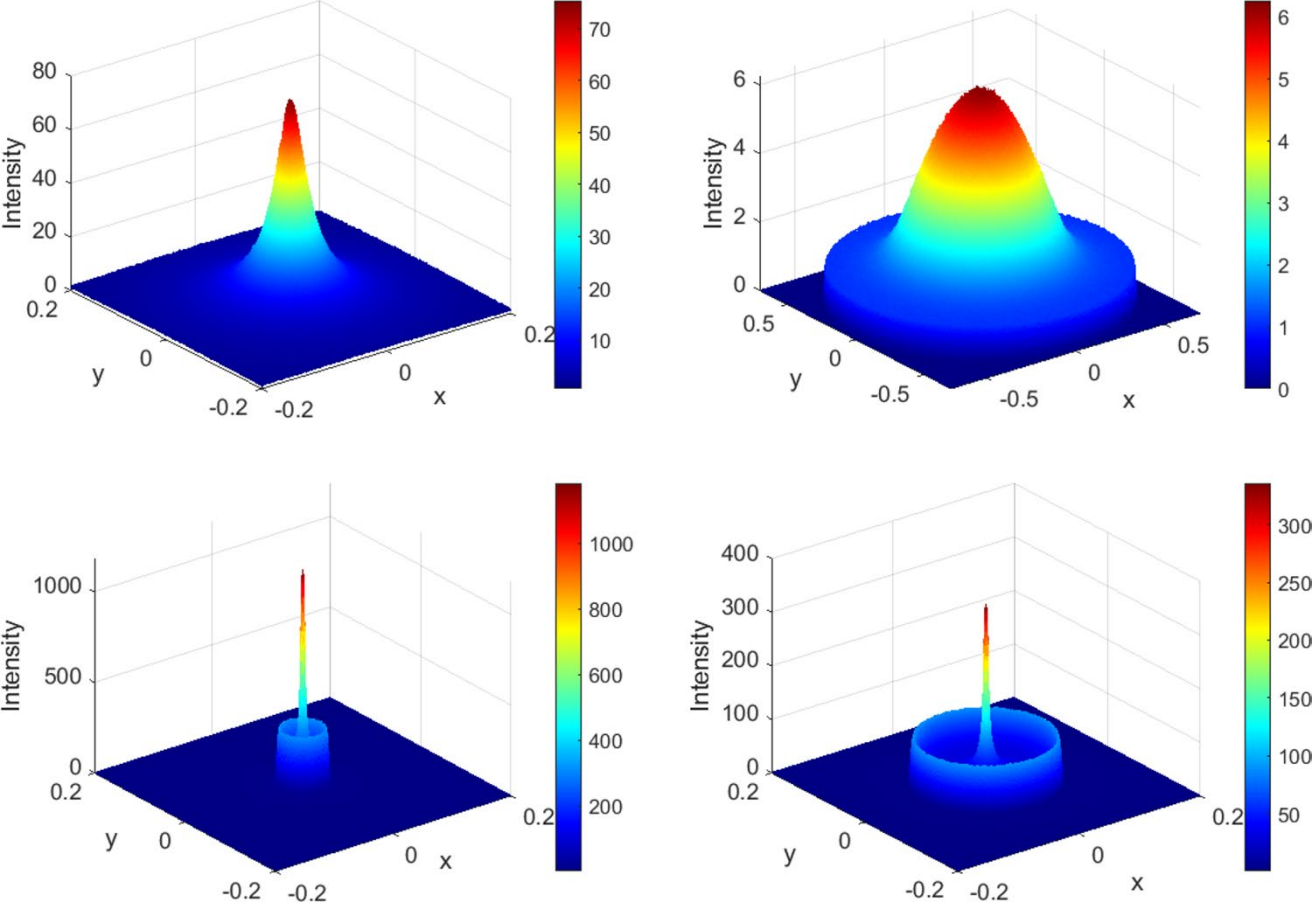
Three dimensional intensity plots, obtained by Monte Carlo ray tracing simulation, with considering the sun finite angular size., on *z* constant surface (colour online). Top left: $z=0.44$ (region I); top right: $z=0.72$ (region III), bottom left: $z=0.57$ (region II) and bottom right: $z=0.63$ (region II). The secondary maxima in region II are noticeable. The figure has been made with *Zemax* 13 R2 SP4 software.

In order to gain a deeper insight, we also carried out Monte Carlo ray tracing simulation to find out how much our previous results will change when the sun finite size is taken into account. Figure 19 exhibits the results of Monte Carlo simulation for the same values of *z* in Fig. 10.

## Summary and conclusion

Solar radiation flux spatial distribution in the interior region of a spherical mirror has been analytically, experimentally and computationally investigated in the framework of geometric optics. A comparison to a paraboloidal dish with the same aperture area was carried out. The aperture diametre of the spherical mirror was chosen at $D=\sqrt{2}R$. In technical terms, the dish rim angle was chosen at $\Phi =\dfrac{\pi }{4}$. It is shown that flux distribution *I*(*r*, *z*) which has cylindrical symmetry crucially depends on *z* the distance of the observation point from the mirror centre. Two types of concentration is shown to occur: point-focus and line-focus. Three distinctive concentration regions on the optical axis have been identified. The concentration is high in the middle region $[R/2,\sqrt{2}R/2]$ whereas the two other regions have a low degree of concentration. In the high concentration region there exists two intensity maxima in the radial direction of the *z* constant plane. The envelope of the reflected rays is also obtained. The second maxima, which occurs on a circle, corresponds to the intersection of *z* constant plane and the envelope. Using beam envelope, the shape of the highly concentrated region is obtained. This shape can significantly help solar thermal engineers to optimally design a cavity receiver and its location. Furthermore, we have evaluated the radiative concentration ratio of the spherical mirror for two types of receivers: flat circular (perpendicular to the optical axis) and vacuum tube line receiver. It is shown that concentration is higher in the line receiver. The distinctive feature of the spherical mirror to the paraboloidal mirror is that the optimal location of the flat receiver depends on its size. The smaller the receiver size, the closer to the quasi focus *R*/2 the optimal location should be. Our findings offer that in a spherical mirror one can alternatively use a line receiver and gains a reasonable thermal energy harvest. What can justify our suggestion is the lower heat loss of the vacuum tube line receivers. The overall efficiency of the dish depends on the interplay of concentration ratio and heat loss amount. Experimental data can reveal which dish can have higher overall efficiency. Work along this experimental line is jointly being considered by a group of CSP researchers.
